# Electroacupuncture Decreases the Leukocyte Infiltration to White Adipose Tissue and Attenuates Inflammatory Response in High Fat Diet-Induced Obesity Rats

**DOI:** 10.1155/2014/473978

**Published:** 2014-08-17

**Authors:** Chorng-Kai Wen, Tzung-Yan Lee

**Affiliations:** ^1^Graduate Institute of Clinical Medicine Sciences, College of Medicine, Chang Gung University, 259 Wen-Hwa 1st Road, Kwei-Shan, Tao-Yuan 333, Taiwan; ^2^Graduate Institute of Traditional Chinese Medicine, College of Medicine, Chang Gung University, 259 Wen-Hwa 1st Road, Kwei-Shan, Tao-Yuan 333, Taiwan

## Abstract

Suppression of white adipose tissue inflammatory signaling may contribute to the pathogenesis of obesity-induced inflammatory response. However, the precise mechanism of efficacy of acupuncture related to adipose tissue remains poorly understood. In the present study we evaluated the anti-inflammatory activities of 10 Hz electroacupuncture (EA) which was applied at the acupoint Zusanli (ST36) for 20 min per day in high-fat diet- (HFD-) induced obesity model. Treatment lasted for one week. Obese rats treated with EA showed significantly reduced body weight compared with the rats in HFD group. EA decreased the number of F4/80 and CD11b-positive macrophages in epididymal adipose tissue. We found that 10 Hz EA given 7 days/week at ST36 acupoints significantly alleviated macrophage recruitment and then improved the obesity-associated factors of sterol regulatory element-binding protein-1 (SREBP-1) and target genes expression in rats with HFD. Adipose tissue inflammatory responses indicated by tumor necrosis factor-*α* (TNF-*α*), IL-6, monocyte chemotactic protein-1 (MCP-1), and CD68 mRNA expression were significantly reduced by EA in obese rats. Additionally, EA was found to significantly reduced serum levels of TNF-*α*, IL-6, and IL-1 in this model. These results indicated that EA improved adipose tissue inflammatory response in obese rats, at least partly, via attenuation of lipogenesis signaling.

## 1. Introduction

Obesity appears to augment the incidence of cardiovascular events and is associated with major risk factors for atherosclerosis, diabetes, metabolic syndrome [1], and coexisting pathological conditions frequently associated with nonalcoholic fatty liver disease (NAFLD) [2].

The decrease in triglycerides (TG) biosynthesis and increase in lipolysis of adipose tissue (AT) lead to elevation in free fatty acids (FFA) in plasma and contribute to ectopic fat deposition in liver and skeletal muscle. The dyslipidemia may promote systemic insulin resistance through several mechanisms, such as induction of oxidative stress and inflammatory responses [3–5]. Obesity is associated with chronic inflammation, evidenced by increased levels of chemokines/cytokines such as monocyte chemoattractant protein-1 (MCP-1), tumor necrosis factor-*α* (TNF-*α*), and increased activation of macrophages in AT [6, 7]. In contrast, obesity is associated with decreased levels of adiponectin, a molecule secreted exclusively by AT with anti-inflammatory properties. In addition, obesity results from an imbalance between fat synthesis (lipogenesis) and fat breakdown (oxidation), which is tightly regulated by the activity of carbohydrate and lipid metabolism. The expression of many lipogenic genes, such as acetyl-CoA carboxylase (ACC) and stearoyl-CoA desaturase (SCD), is transcriptionally regulated by sterol regulatory element-binding protein-1 (SREBP-1) and then activates expression of the target gene in obesity [8].

Acupuncture, one of the oldest healing practices, represents the most rapidly growing complementary therapy which is recognized by both the National Institutes of Health and the WHO. Both experimental and clinical current data suggest that acupuncture exerts beneficial effects on the mechanisms of obesity and obesity-related insulin resistance [9–11]. It would be desirable to control obesity by safe and effective treatment modalities, and among the different methods acupuncture is one of the most popular complementary treatments. Chronic inflammation plays a critical role in the pathogenesis of obesity and diabetes [12] and may be an important link between obesity and the related disorders of metabolic disease. Various modalities of weight loss used to treat obesity have been shown to reduce levels of proinflammatory molecules in obese subjects [13, 14], indicating that weight loss may exert beneficial effects by reducing the underlying inflammation.

In clinical studies, acupuncture induced a potential weight loss effect and produced greater improvements in metabolic parameters in obese patients. Nevertheless, the effect of acupuncture on inflammatory response associated with diet-induced obesity remains to be elucidated. Furthermore, it is not clear whether 10 Hz electroacupuncture had a weight loss effect, when EA is given more frequently in rats with HFD-induced obesity. In this study, we test the hypothesis that 10 Hz EA, given 7 days/week for an intense stimulation, is enough to examine the effect of EA on obesity-linked inflammation in AT and blood.

## 2. Methods

### 2.1. Obesity Induction and Electroacupuncture Treatments

Male SD rats (130 ± 12 g) were obtained from the Taiwan NSC Animal Center at 4 weeks of age. After a 1-week acclimation period, control group with normal chow and the rest of rats were switched to high fat diet for 15 weeks to induce obesity and then were randomly divided into three groups: the animals fed only with the high fat diet (B), the high fat diet with three times of electroacupuncture treatment (C), and the high fat diet with seven times of electroacupuncture treatment (D) groups. All groups were allowed to eat* ad libitum*, and food intake and body weight were recorded once/week for the duration of the study. Age-matched normal animals were maintained on regular chow (Laboratory Autoclavable Rodent Diet 5010, Purina Mills Inc., Richmond, IN) which provided 28% Kcal from protein; 59% Kcal from carbohydrate; and 13% Kcal from fat; the high fat diet (Mouse Diet High Carbohydrate High Fat F3282, BioServ, Frenchtown, NJ), which contained 59% calories in fat. In terms of weight, the total fat was 35.8% (wt/wt) in the diet. There was 33.4% saturated, 0.86% monounsaturated, and 1.54% polyunsaturated fatty acids in the diet (59% Kcal from fat; 16% Kcal from protein; 24% Kcal from carbohydrate). Animal experiment protocol has been reviewed by the Institutional Animal Care and Use Committee of Chang Gung University (IACUC Approval number CGU12-135), and the Committee recognizes that the proposed animal experiment follows the guideline as shown in the* Guide for Laboratory Animal Facilities and Care* as promulgated by the Council of Agriculture, Executive Yuan, China.

Low-frequency EA (10 Hz) was performed to conscious rats once per day for 3 or 7 consecutive days. Electroacupuncture (EA) was applied at the acupoint Zusanli (ST36) for 20 min per day, with seven treatments being performed. Treatment lasted for one week. ST36 located at the anterior tibia muscle near knees were identified based on previous studies [10, 15, 16]. After adjusting the EA apparatus to 10 Hz (Digitimer DS3 stimulator, Letchworth Garden City, UK), the needles (0.5 in., 32 gauge) were inserted to depths of 0.3-0.4 cm into the muscle layer of the selected acupoints. The intensity varied from 0.5 to 1.0 mA during stimulation and was adjusted to produce local muscle contraction. Before needle insertion, the rats were lightly anesthetized with isoflurane (2% in 1 : 1 mixture of oxygen and air) for about 2-3 min. The positively charged (red) clip was connected to the right needle and the negatively charged (black) clip was connected to the left needle. All animals were fasted overnight; each experimental group consisted of 5 animals were sacrificed by CO_2_ asphyxiation and were decapitated at specific time points in the morning. The trunk blood of each rat was collected separately, and the serum was stored at −20°C until assayed.

### 2.2. Biochemical Measurement and Histological and Immunohistochemical Analyses

Epididymal fat pads and blood samples were harvested immediately after euthanization, weighed, flash frozen in liquid nitrogen, and stored at −80°C. Adipose tissue was fixed in 10% formalin, embedded in paraffin, cut into 5 *μ*m thick sections, and stained with hematoxylin and eosin (H&E). Staining was performed using standard techniques by the Pathology Core of the Research Center at the Chang Gung Memorial Hospital and examined under a light microscopy by an experienced pathologist. Computer assisted morphometry was used to determine adipocyte areas (Olympus image system software) and approximately 2000 cells were evaluated. Immunostaining for F4/80, CD11b+ (BioLegend, San Diego, CA), CD36, and neutrophil (Abcam, Cambridge, MA) was performed in sections using the specific antibodies and an avidin-biotin complex immunoperoxidase method. To determine the serum alanine aminotransferase (ALT) levels, commercial kits (Roche Diagnostics GmbH, Manheim, Germany) were used. The concentrations of serum tumor necrosis factor-*α* (TNF-*α*), interleukin-1 (IL-1), and interleukin6 (IL-6) were determined using sandwich ELISA. The capture and detection antibodies against rat TNF-*α*, IL-1, and IL-6 were purchased from R&D systems (Minneapolis, MN). The concentrations of TC, TG, and free fatty acid (FFA) were determined using a commercial Quantification kit (Randox, Antrim, UK). Plasma glucose concentration was determined with the use of a glucose analyser (Yellow Springs Instruments 2300, Yellow Springs, OH). Insulin concentrations were measured with commercially available RIA kits (Medicorp, Montréal, PQ, Canada; ICN Pharmaceuticals).

### 2.3. Quantitative Immunohistochemistry

To quantify the number of cells stained with related inflammatory factors in adipose tissue, three sections were stained with monoclonal antibody against mouse F4/80, CD11b+, and neutrophil, respectively. Images were extracted from scanned slides using image analysis software (OLYMPUS Xcellence real-time imaging system). The average number of index-positive cells/40x field was quantified from five fields/rat/time point.

### 2.4. Real-Time Polymerase Chain Reaction (PCR) Analysis

Total RNA was extracted from the epididymal fat tissue using the guanidinium-phenol-chloroform method. Total RNA (5 *μ*g) was reverse-transcribed using the RevertAid First Strand cDNA Synthesis kit according to the manufacturer's instructions. The cDNA was amplified using the TaqDNA polymerase kit (Fermentas, Vilnius, Lithuania). Real-time PCR was performed on a LightCycler 1.5 apparatus (Roche Diagnostics GmbH) using the LightCycler FastStart DNA MasterPLUS SYBR-Green I kit according to the manufacturer's protocol. Direct detection of PCR products were monitored by measuring the fluorescence produced by the result of TaqMan probe hydrolysis after every cycle. For both TaqMan and SYBR Green methods amplification efficiencies were tested for the gene of interest (GOI) and housekeeping gene. All samples were tested with the reference gene GAPDH for data normalization to correct for variations in RNA quality and quantity. All samples were performed in triplicate. These measurements were then plotted against cycle numbers. The parameter threshold cycle (Ct) defined as the cycle number at which the first detectable fluorescence increase above the threshold observed. For fold-changes calculation in relative gene expression, equation ΔCt, where ΔCt = Ct (GOI) − Ct (GAPDH) was used.

### 2.5. Data Analysis

Data were presented as means ± SEM. The statistical analyses were performed using a one-way analysis of variance followed by the Student Newman-Keuls multiple-range test. A value of *P* < 0.05 was considered to indicate a statistically significant difference.

## 3. Results

### 3.1. Effect of EA on Body Weight and Aminotransferase Levels

**Figure 1:**
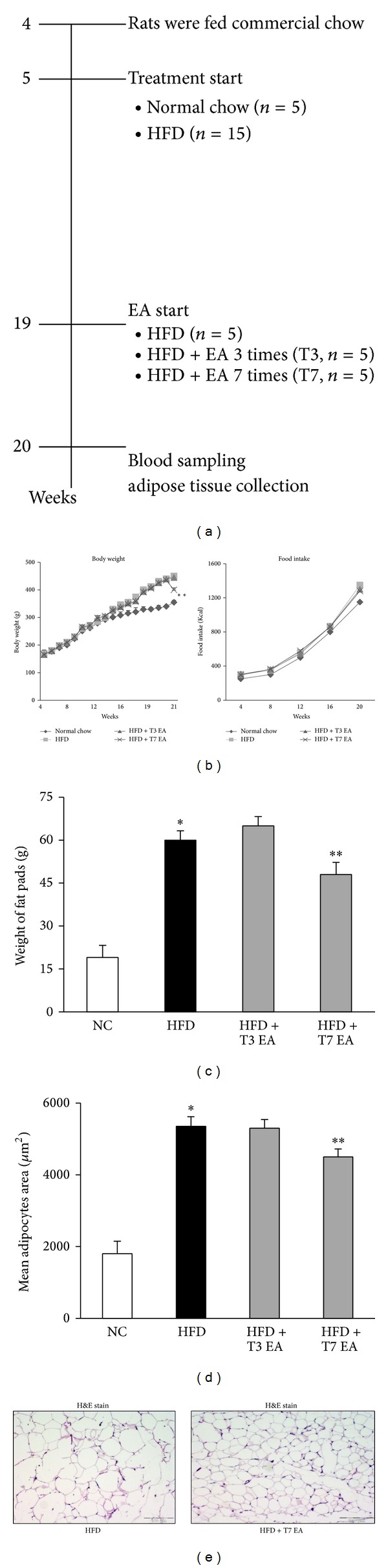
Effects of electroacupuncture on body weight, fat accumulation, and adipocyte hypertrophy in high fat diet rats. Animals with high fat diet-induced obesity were treated with EA once by daily for three times (T3) or seven times (T7) in the age of 20 weeks. Pair-feeding was conducted in three separate groups of obese rats to achieve the same body weight during this period; *n* = 5 rat/group. Flow chart of the study design (a), detailed view of the growth curve during the experimental period, body weight (b, left panel), and food intake was measured on a per-cage basis throughout the study and represents cumulative energy intake (b, right panel) and total adipocyte size expressed at the end of treatment (c). H&E staining showing a decrease in adipocyte size in EA treatment rats, compared with obese animals fed a HFD (e). Distribution of adipocyte sizes indicates a shift in the size of the adipocyte population toward larger hypertrophied cells, reflected in a significant increase in the mean adipocyte size in HFD animals. *P < 0.05, compared with normal chow group; **P < 0.05, compared with HFD animals. NC: normal chow. HFD: high fat diet. EA: electroacupuncture. T3: three times of EA treatment. T7: seven times of EA treatment. Original magnification ×40. Scale bars, 100 *μ*m.

Treatment with T7EA almost abolished the high fat diet-induced epididymal and perirenal fat (Table 1) accumulation in parallel with the body weight reduction. In keeping with this data, serum liver enzymes measured as an indication of liver function followed a similar pattern. Normal chow feeding had no significant effect on liver function in rats. Serum alanine transaminase (ALT) levels were significantly raised in HFD obese animals, suggesting hepatocyte damage. EA treatment reduced ALT levels in HFD rats and tended to reduce aspartate transaminase levels (AST) as well. EA significantly lowered AST and ALT levels, though they remained above normal levels (Table 1). Intense EA treatment of obese rats (HFD + T7 EA) markedly induced weight loss during the first week as compared with obese controls (HFD group). However, the body weight of HFD + T3 EA rats remained higher than that of normal chow controls (Figure 1(b), left panel). Food intake did not significantly differ among the high fat diet group and EA treated groups (Figure 1(b), right panel). After seven times of EA treatment, obese animals had lower body weight; fat pads were also smaller than vehicle control (Figure 1(c)), although both groups consumed the same amount of HFD (Figure 1). EA treatment rats had smaller adipocytes than HFD rats (Figures 1(d) and 1(e)).

### 3.2. EA Decreases mRNA Expression of Lipogenic Genes

**Figure 2:**
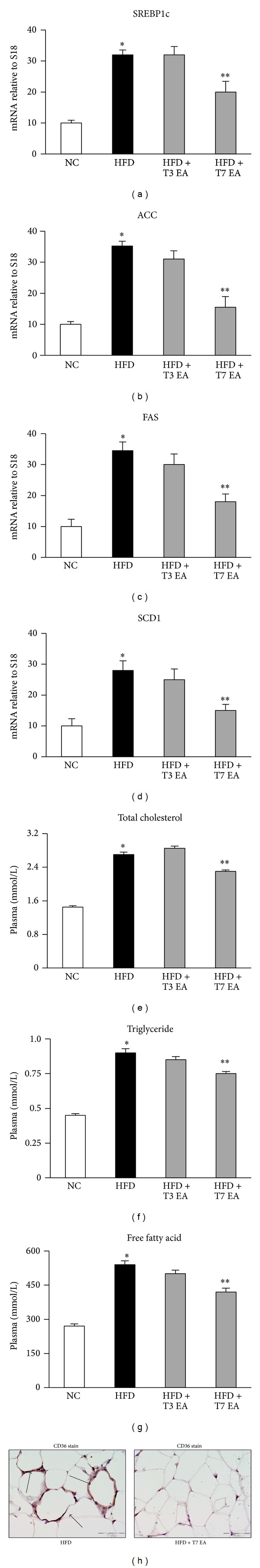
Effects of electroacupuncture on adipose tissue expression of lipogenic genes and serum lipid profiles. mRNA levels of selected lipogenic genes in adipose tissue after EA treatment were examined by quantitative reverse transcription polymerase chain reaction. Each result represented the experiment performed in triplicate assays in the different experimental groups. Values are expressed as means ± SEM. *P < 0.05, compared with normal chow group; **P < 0.05, compared with HFD animals. NC: normal chow. HFD: high fat diet. EA: electroacupuncture. T3: three times of EA treatment. T7: seven times of EA treatment. SREBP1c: sterol response element binding protein 1c. ACC: acetyl-CoA carboxylase. FAS: fatty acid synthase. SCD-1: stearoyl-CoA desaturase-1. Original magnification ×40. Scale bars, 100 *μ*m.

To elucidate the underlying mechanisms of the effects of EA, we used quantitative RT-PCR after 20 weeks of HFD feeding to examine EA-induced early changes in lipogenesis gene expression in adipose tissues (Figure 2). Intense treatment with EA significantly downregulated the mRNA levels of SREBP1c, FAS, ACC1, and SCD1, which are lipogenic enzymes in the adipose tissue, compared with the levels in the high fat vehicle group (Figures 2(a)–2(d)). The mRNA level of SREBP1c, the master regulator of fatty acid synthesis, was lower in the EA group than in the HFD group. Similarly, EA treatment decreased plasma lipid profiles, including total cholesterol, triglyceride, and free fatty acids (Figures 2(e)–2(g)) in HFD groups. On the other hand, there were marked differences in the immunohistochemical analysis that fatty acid transporter (CD36, Figure 2(h)) between the groups in epididymal white adipose tissue.

### 3.3. Effect of EA on Adipose Tissue Inflammation

**Figure 3:**
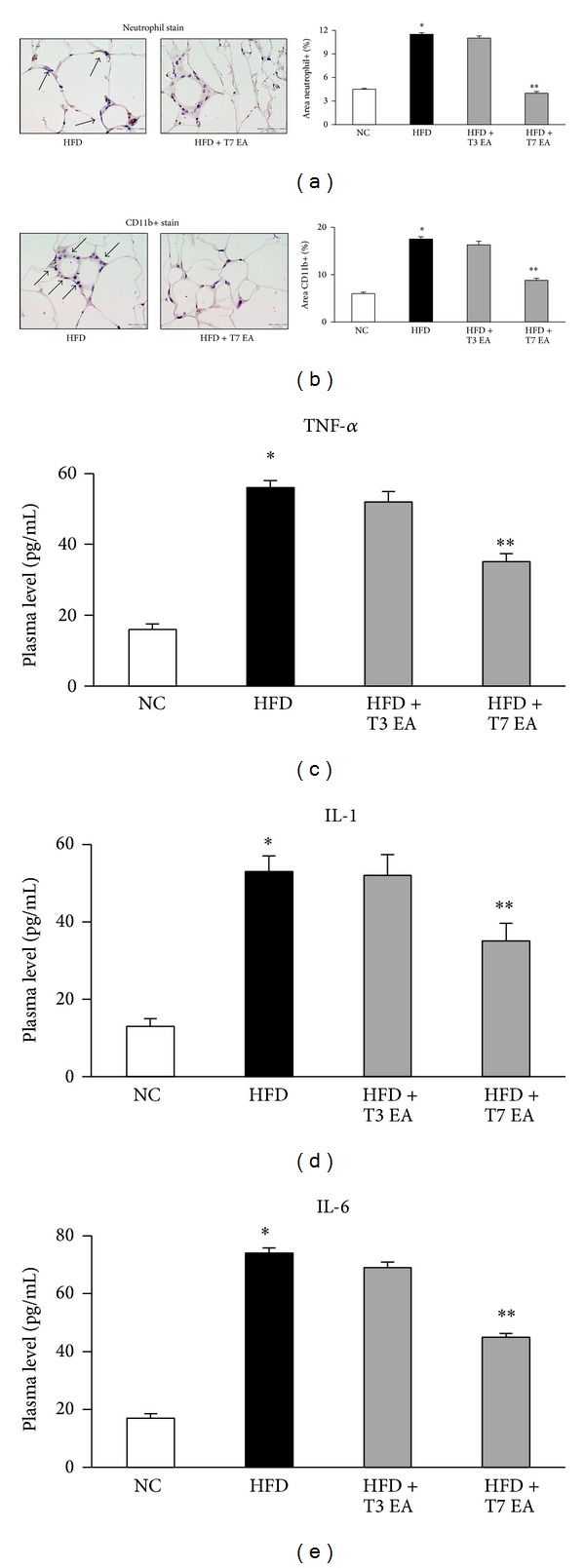
Effects of electroacupuncture on both neutrophil and macrophage activation markers in adipose tissue and cytokines levels altered in animals with dietary obesity. Neutrophil (a), CD11b+ (b) in adipose tissue, and serum TNF-*α* (c), IL-1 (d) and IL-6 (e) levels, in rats fed with either an HFD or a normal chow. Each result represented the experiment performed in triplicate assays in the different experimental groups. Arrows indicate neutrophil and CD11b+ positive cells, respectively. Results are expressed as means ± SEM. *P < 0.05, compared with normal chow group; **P < 0.05, compared with HFD animals. NC: normal chow. HFD: high fat diet. EA: electroacupuncture. T3: three times of EA treatment. T7: seven times of EA treatment. Original magnification ×40. Scale bars, 100 *μ*m.

Because obesity is associated with leukocyte accumulation in adipose tissue, which may be mediated by cytokines and contribute to adipose tissue inflammation, we examined effects of EA on neutrophil accumulation in adipose tissue. Compared to normal chow rats, HFD animals had increased levels of neutrophile (Figure 3(a)) and CD11b+ (activation of macrophage marker, Figure 3(b)) in adipose tissue. Compared to HFD group, HFD rats treated with intense EA have significantly different levels of neutrophil/macrophage cell markers. Based on the active roles of cytokines in inflammation, we examined the effect of EA on proinflammatory cytokines genes expression in adipose tissue. Compared to the lean control, obese HFD rats had increased levels of several cytokines, including TNF-*α*, IL-1, and IL-6, in adipose tissue (Figures 3(c)–3(e)). EA treated animals also had lower mRNA levels of TNF-*α* and IL-6 than HFD group.

### 3.4. EA Decreases Macrophage Infiltration into Adipose Tissue

**Figure 4:**
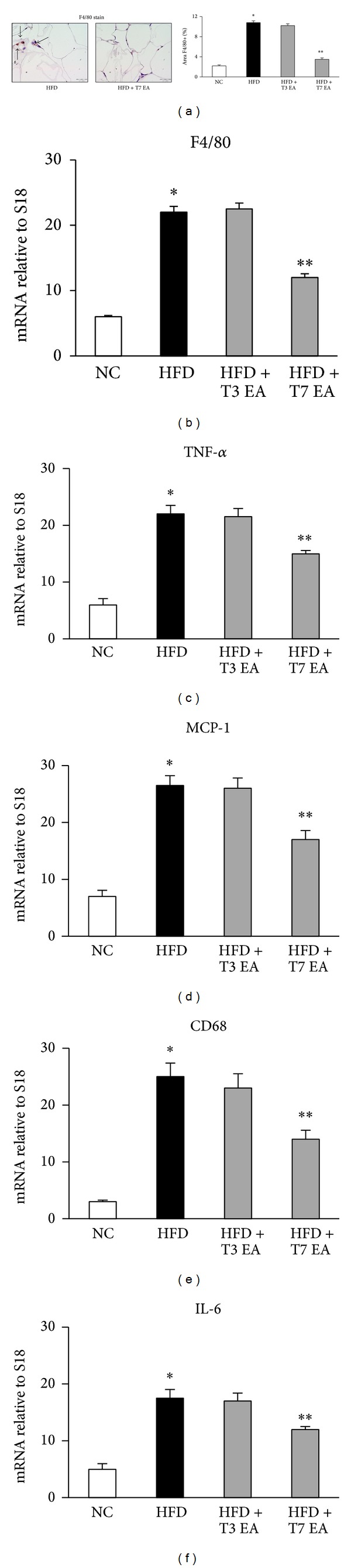
Effects of electroacupuncture on adipose tissue macrophage cell marker (F4/80) and inflammation mRNA levels of chemokines in animals with dietary obesity. Histomorphometrical analysis of adipose tissue sections stained with F4/80 (original magnification ×40) (a) and mRNA quantitated in rats adipose tissue by quantitative reverse transcription polymerase chain reaction. Each result represented the experiment performed in triplicate assays in the different experimental groups. Arrows indicate F4/80 positive cells. Results are expressed as means ± SEM. *P < 0.05, compared with normal chow group; **P < 0.05, compared with HFD animals. NC: normal chow. HFD: high fat diet. EA: electroacupuncture. T3: three times of EA treatment. T7: seven times of EA treatment. Scale bars, 100 *μ*m.

Increased adiposity is associated with increased infiltration of macrophages into the adipose tissue. Therefore, we determined the effect of EA on inflammatory responses in epididymal adipose tissue. Consistent with previous result, staining of adipose tissue with antibodies to the macrophage marker F4/80 (Figure 4(a)) showed a high frequency of macrophages in the adipose tissue of HFD rats, which was confirmed by gene expression analysis of F4/80 with real-time PCR (Figure 4(b)). The number of F4/80 positive cells in adipose tissue was significantly different among HFD and HFD with EA treatment groups. EA treatment in HFD rats significantly decreased adipose tissue TNF-*α* (Figure 4(c)), MCP-1 (Figure 4(d)), CD68 (Figure 4(e)), and IL-6 (Figure 4(f)) mRNA levels compared to those in HFD controls. In the adipose tissue of rats treated with EA, however, the number of macrophages was decreased concomitant with decreased expression of F4/80 and CD68 mRNA. Consistent with the increased macrophage content, the expression of MCP-1, a chemokine involved in macrophage recruitment, was upregulated nearly threefold by the HFD.

## 4. Discussion

The aim of this study was to address the hypothesis that EA treatment markedly reduced fat mass in obese animals, with the protection from the inflammatory response to obesity in the diet-intake-matched control. The decreases in inflammation with intense EA treatment may contribute to its beneficial effects on metabolic parameters associated with diet-induced obesity.

### 4.1. Electroacupuncture Decreased Inflammatory Response on Adipose Tissue

Although weight loss and hypoglycemic effects of EA have been documented [9–11, 15, 16], most of the previous studies did not show that EA treatment correlated with inhibition of inflammatory response. Intense EA treatment of obese mice decreased fat mass as compared to obese vehicle. EA may decrease fat mass through increasing whole body energy expenditure and decreasing lipogenesis in adipose tissue of obese rats, which may also contribute to inflammatory response reduction in EA-treated rats. Obesity is associated with adipose tissue inflammation characterized by increased chemokines/cytokines and increased leukocyte accumulation in adipose tissue [17, 18]. EA treatment of obese mice decreased inflammatory response in adipose tissue primarily by decreasing MCP-1, a chemokine critically involved in leukocyte migration [19]. Indeed, adipose tissue CD11b+ cells, which are increased and show proinflammatory characteristics in obesity [20], were reduced with EA treatment. Current study found that EA treatment of obese animals also decreased TNF-*α* expression in adipose tissue as compared with obese controls. These inflammatory changes are caused in part by the infiltration of macrophages and other immune cells into white adipose tissue and have been seen in both rodents and humans [6, 21–23].

### 4.2. Electroacupuncture Inhibits Adipose Tissue Macrophage Infiltration and Chemokines Levels in Animals with Dietary Obesity

Adipocyte hypertrophy, adipocyte apoptosis/necrosis, and local increases in free-fatty acids (FFAs) have been implicated in potentiating adipose tissue inflammation [9]. The initial stimulus for the influx of the immune cells into adipose tissue may be complex, but elevated levels of the MCP-1 in adipose tissue are thought to play a role [22]. Changes in CD68 mRNA were used as a marker of macrophage infiltration throughout this study. Although CD68 is not exclusively expressed on macrophages (it is also expressed on neutrophils, basophils, and large lymphocytes), CD68 mRNA has been shown to increase in adipose tissue of obese mice to a similar degree as F4/80, another commonly used marker of macrophages [24]. F4/80 immunohistochemistry appeared to confirm the results from CD68 quantitative real-time PCR, demonstrating a lack of macrophage infiltration in obese treated with EA when animals were fed on HFD. Current data showing that macrophages are present in adipose tissue of rat fed a HFD demonstrate that the signals for macrophage infiltration into white adipose tissue are also multifactorial. Interestingly, our data indicate that the obesity-associated increase in adipose macrophages can be prevented by the intense EA. Collectively, these results demonstrate that EA treatment which reduces total adipose macrophage infiltration, thereby reducing the percentage of macrophage number, a predominant cellular source of inflammatory adipokines, may explain the reduced mRNA levels of IL-6 and MCP-1 in the HFD group. Overall, the EA treatment dramatically ameliorated the adipose tissue inflammatory phenotype, including reducing (i) circulating levels of adipocyte derived inflammatory hormones, (ii) adipose inflammatory cytokine mRNA expression, and (iii) total macrophage infiltration and the percentage of inflammatory cells.

### 4.3. Electroacupuncture Suppression of Lipogenic Genes and Serum Lipid Profiles on Adipose Tissue

EA downregulated lipogenic genes, such as FAS, ACC, and SCD1 expression in the adipose tissue in obese animals. Several lines of evidence indicate that the suppression or disruption of ACC, a cytosolic enzyme that catalyzes the carboxylation of acetyl-CoA to form malonyl-CoA, leads to the reduction of hepatic triglyceride synthesis and accumulation [25], suggesting that suppression of ACC expression could contribute to reducing body fat accumulation. SCD1, which catalyzes the biosynthesis of monounsaturated fatty acids from saturated fatty acids, also has an important role in energy metabolism and body weight regulation [26]. The expression of several lipogenic genes is regulated by SREBP-1c at the transcriptional level. EA decreased SREBP-1c mRNA levels in the adipose tissue of obese rats. In addition, EA treatment decreased the expression of lipogenic genes along with a decrease in SREBP-1c mRNA. Further, in our preliminary studies, EA also reduced SREBP-1c and FAS mRNA expression in the liver under the HFD condition (data not shown). Overall, the results suggest that EA suppresses the lipogenic pathway by downregulating SREBP-1c, which leads to a reduced accumulation of body fat.

Histological analysis of the adipose tissue revealed that EA treatment animals had smaller adipocytes than HFD-induced obese animals without EA, which correlated with a lower number of infiltrating macrophages. Obesity-associated low-grade inflammation characterized by an increased abundance of macrophages in the adipose tissue is recognized as a key step in the pathogenesis of insulin resistance [27]. Therefore, EA may be useful as a potential countermeasure against obesity and type 2 diabetes. Since the low-grade chronic inflammation associated with obesity further complicates many disease states, elucidation of mechanisms through which EA treatment impacts the clinical outcome of concurrent diet-induced obesity has translational utility. Moreover, obesity worsens the clinical outcome of various diseases wherein adipose-derived inflammatory mediators perpetuate inflammatory responses, promoting a more aggressive clinical course. Focusing on the major tissue sites, namely, the visceral adipose tissue (local site of obesity-associated inflammation and a major endocrine organ), we were able to document changes in the local and systemic inflammatory milieu while tracking changes in macrophage populations driving the obesity-associated inflammatory response after EA treatment.

Acupuncture is a form of sensory stimulation in which thin needles are placed in the skin and muscle. That has raised several intrinsic challenges that are unique to its methodology: nonstandardized or individualized techniques and appropriate controls. When a nonacupuncture point is used, that means a needle is applied to the same depth and for the same duration as the treatment group but in a location that has no known effect. In addition, the animal legs are too thin to accurately make needles able to be inserted in appropriate nonacupoint. This factor also contributed to the limitations of our experiments. Another limitation of this study is that EA stimulation at ST36 can regulate body weight independent of food intake, presumably by modulating fat pads storage in the periphery. We are not certain whether food intake inhibition is the only cause of body weight reduction. Whether EA also regulates energy expenditure is unknown. Future studies should be carried out to determine whether stimulation of ST36 acts through the potential mechanism to impact food intake and body weight regulation.

## 5. Conclusions

In summary, we established model of high fat diet-induced obesity to demonstrate the ability of intense effects of low-frequency EA to ameliorate obesity progression, in part, by reducing inflammatory cytokine gene expression and immune cell populations in adipose tissue.

## Figures and Tables

**Table 1:** Plasma biochemistry and fat pad weights in obese rats and with electroacupuncture treatment.

Groups	Normal chow rat (*N* = 5)	HFD obese rat (*N* = 5)	HFD + EA	
			T3 (*N* = 5)	T7 (*N* = 5)
ALT (IU/dL)	45 ± 5	90 ± 6∗	88 ± 7	58 ± 4∗∗
AST (IU/dL)	59 ± 9	142 ± 9∗	137 ± 5	82 ± 8∗∗
Bilirubin (mg/dL)	0.5 ± 0.1	0.5 ± 0.1	0.5 ± 0.1	0.5 ± 0.1
Epididymal fat pad (g/100 g BW)	2.3 ± 0.6	3.2 ± 0.6∗	3.2 ± 1.6	2.9 ± 0.8∗∗
Perirenal fat pad (g/100 g BW)	4.8 ± 1.3	6.7 ± 2.5∗	6.5 ± 2.3	5.6 ± 2.0∗∗

(1) Mean ± SEM.

(2) Statistical significance of experimental factors was calculated using one-way ANOVA.

(3) Values are significantly different at *P* < 0.05. *P < 0.05 versus normal diet rats; **P < 0.05 versus high fat diet rats. HFD: high fat diet, EA: electroacupuncture, T3: three times of EA treatment, and T7: seven times of EA treatment.
